# Long non-coding RNA *SPRY4-IT1* promotes epithelial–mesenchymal transition of cervical cancer by regulating the miR-101-3p/ZEB1 axis

**DOI:** 10.1042/BSR20181339

**Published:** 2019-06-04

**Authors:** Ming-Jun Fan, Yong-Hui Zou, Peng-Juan He, Shuai Zhang, Xiao-Mei Sun, Chang-Zhong Li

**Affiliations:** 1 Department of Obstetrics and Gynecology, Shandong Provincial Hospital Affiliated to Shandong University, Jinan 250021, P.R. China; 2 Department of Obstetrics and Gynecology, First Clinical Medical College, Shandong University of Traditional Chinese Medicine, Jinan 250021, P.R. China

**Keywords:** cervical Cancer, EMT, Long non-coding RNA, metastasis, miR-101-3p, SPRY4-IT1

## Abstract

Background: Emerging evidences have indicated that long non-coding RNAs (LncRNAs) play vital roles in cancer development and progression. Previous studies have suggested that overexpression of SPRY4 intronic transcript 1 (*SPRY4-IT1*) predicates poor prognosis and promotes tumor progress in cervical cancer (CC). However, the underlying mechanism of *SPRY4-IT1* in CC remains unknown. The aim of the present study is to evaluate the function and mechanism of *SPRY4-IT1* in CC.

Methods: *SPRY4-IT1* was detected by quantitative PCR. Wound-healing assay and Transwell assay were performed to detect cell migration and invasion, respectively. Western blotting assays were used to analyze the protein expression of E-cadherin, N-cadherin and vimentin. Tumor xenografts experiments were performed to detect the effect of *SPRY4-IT1 in vivo.* Dual luciferase reporter assay was used to investigate potential molecular mechanism of *SPRY4-IT1* in CC cells.

Results: *SPRY4-IT1* was up-regulated in CC cell lines. Knockdown of *SPRY4-IT1* significantly inhibited CC cells migration and invasion *in vitro* and *in vivo*. Moreover, knockdown of *SPRY4-IT1* significantly suppressed the epithelial–mesenchymal transition (EMT) of CC by increased E-cadherin expression and decreased the N-cadherin and vimentin expression. Mechanically, *SPRY4-IT1* could directly bind to *miR-101-3p* and effectively act as a competing endogenous RNA (ceRNA) for *miR-101-3p* to regulate the expression of the target gene *ZEB1*.

Conclusions: Our findings indicate that the *SPYR4-IT1/miR-101-3p*/ZEB1 axis contributes to CC migration and invasion, which may provide novel insights into the function of lncRNA-driven tumorigenesis of CC.

## Introduction

Cervical cancer (CC) is the fourth most commonly diagnosed cancer and the fourth leading cause of cancer-related deaths among females worldwide [1]. With the improvement of diagnostic techniques and therapeutic strategies, the incidence and mortality rates of CC has decreased [2]. However, the overall prognosis of CC patients still remains poor, especially in developing countries [3]. Tumor invasion and metastasis are key processes affecting the efficacy and prognosis of CC [4]. Therefore, exploring ways to inhibit these processes has important implications for improving the prognosis and quality of life of patients with CC.

Epithelial–mesenchymal transition (EMT) defines an orchestrated series of transcriptional and morphological program during which cells lose their typical epithelial characteristics and acquire mesenchymal properties [5]. Recent studies have suggested that EMT may play an important role in epithelial cancer progression and metastasis including cervical carcinoma, which have dramatic phenotypic changes by the loss of epithelial marker proteins such as E-cadherin and the acquisition of mesenchymal marker protein such as vimentin [6,7]. EMT is thought to be critical for the initial transformation from benign to invasive carcinoma, whereas MET (the reverse of EMT) is critical for the later stages of metastasis [8]. Just as a critical EMT event is the down-regulation or silencing of E-cadherin, the re-expression of E-cadherin is proposed to be the important hallmark of MET [9], thereby representing a potently novel strategy for development of new methods to improve therapeutic interventions of CC.

ZEB1 is increasingly recognized as a central regulator of EMT and invasion in solid tumors through transcriptional repression of *E-cadherin* gene via direct interaction with its E-boxes [10–12]. Increased ZEB1 expression has been reported in CC tissue, indicating that ZEB1 expression may be associated with progression of CC [13]. Importantly, up-regulation of ZEB1 at the post-transcriptional level could be induced by the loss or repression of microRNAs (miRNAs) that selectively target ZEB1 in cancer [5]. MiR-101, one of these potential miRNAs, is negatively regulated in different types of cancers including CC and considered to be a tumor suppressor [14–16]. While miR-101 expression was shown to correlate with ZEB1 signaling in breast cancer cells [16], little is known about the role of miR-101/ZEB1 signaling in regulating the EMT process of CC.

Accumulating evidence has demonstrated that long non-coding RNAs (lncRNAs) play a non-negligible role in tumorigenesis [17], and a new post-transcriptional regulatory mechanism that LncRNAs can function as a natural miRNA sponge has been recently revealed [18]. For example, *MALAT1* mediates Rac1 expression by acting as an *miR-101b* sponge [19]. LncRNA Unigene56159 acts as a sponge for *miR-140-5p* to modulate ZEB2 expression in hepatocellular carcinoma [20]. SPRY4 intronic transcript 1 (*SPRY4-IT1*), an lncRNA derived from an intron within *SPRY4* gene, has been recently revealed as oncogenic regulatory hubs or tumor suppressors in different cancers. For example, *SPRY4-IT1* was down-regulated in gastric cancer and contributed to gastric cancer cells metastasis partly via regulating the EMT process [21]. By contrast, it was reported to promote metastasis of bladder cancer and colorectal cancer by targeting *miR-101*, and knockdown of its expression inhibited cell growth, invasion and induced cell apoptosis [22,23]. A previous study found that *SPRY4-IT1* was up-regulated in CC [24], whereas its function and mechanism of action is not well documented.

In the present study, we explore the biological roles of *SPRY4-IT1* on the phenotypes of CC cells both *in vitro* and *in vivo*. Furthermore, mechanistic analysis reveals that *SPRY4-IT1* functions as a miRNA sponge to positively regulate the expression of ZEB1 through sponging *miR-101-3p*, thus playing an oncogenic role in CC pathogenesis. Together, our study elucidates the role of LncRNA *SPRY4-IT1* as regulations of CC progression, and sheds new light on LncRNA-directed diagnostics and therapeutics in CC.

## Materials and methods

### Cell culture

The CC cell lines (HeLa and CaSki) were purchased from American Type Culture Collection (ATCC, Manassas VA, U.S.A.). Cells were cultured in Dulbecco’s modified Eagle’s medium (DMEM)/F12 supplemented with 10% fetal bovine serum (FBS), 100 U/ml penicillin, and 100 mg/ml streptomycin (Gibco) and maintained in humidified air at 37°C with 5% CO_2_ atmosphere.

### DNA constructs and cell transfection

Short hairpin RNA (shRNA) against *SPRY4-IT1* (sh-SPRY4-IT1: 5′-TGCTTTATCTGTAGGACAT-3′) and negative control shRNA (sh-NC: 5′-GTTCTCCGAACGTGTCACGT-3′) were synthesized by Genema (Shanghai, China). *miR-101-3p* mimics (sense: 5′-UACAGUACUGUGAUAACUGAA-3′; antisense: 5′-UUCAGUUAUCACAGUACUGUA-3′), *miR-101-3p* inhibitor (5′-UUCAGUUAUCACAGUACUGUA-3′) and plasmids were purchased from Genema (Shanghai, China). The ZEB1 full-length was cloned into pcDNA3.1 plasmid. And human ZEB1 3′-untranslated region (UTR) fragment and *SPRY4-IT1* gene containing putative binding sites for *miR-101-3p* reporter vector were synthesized by Genechem from RiboBio.

HeLa or CaSki cells (5 × 10^5^) were planted in six-well plates 24 h prior to transfection with shRNAs, *miR-101-3p* mimics, *miR-101-3p* inhibitor, and pcDNA3.1-ZEB1 with 60–70% confluence, then transfected with Lipofectamine 2000 (Invitrogen, Carlsbad, CA, U.S.A.) according to the manufacturer’s instructions. The transfected cells were harvested at 48 h after transfection.

### RNA extraction, reverse transcription and quantitative PCR

Total RNA was isolated with TRIzol reagent (Invitrogen) according to the manufacturer’s instructions. Complementary DNA was synthesized with random primers using a reverse transcription kit PrimeScript RT reagent Kit (Takara Biomedical Technology, Dalian, China) or commercial miRNA reverse transcription PCR kit (RiboBio). Quantitative real-time PCR (qPCR) analysis was carried out using the SYBR Premix Ex Taq kit (Takara Biomedical Technology). The primer set for *SPRY4-IT1* was 5′-AATATGCCCAGTGGAGCCAT-3′ (forward) and 5′- GGCCTTGGAATCAGAAAGCA-3′ (reverse). The primer set for *miR-101-3p* was 5′-GCGCGCATACAGTACTGTGATA-3′ (forward) and 5′- CGGCCCAGTGTTCAGACTAC-3′ (reverse). The primer set for *ZEB1* was 5′-TATGAATGCCCAAACTGCAA-3′ (forward) and 5′-TGGTGATGCTGAAAGAGACG-3′ (reverse). The primer set for glyceraldehyde-3-phosphate dehydrogenase (GAPDH) was 5′-CCAGGTGGTCTCCTCTGA-3′ (forward) and 5′-GCTGTAGCCAAATCGTTGT-3′ (reverse). The primer set for U6 was 5′-CTCGCTTCGGCAGCACA-3′ (forward) and 5′-AACGCTTCACGAATTTGCGT-3′ (reverse). All data analyses were operated using the StepOnePlus Real-Time PCR System (Applied Biosystems, Foster City, CA, U.S.A.). All reactions were run in triplicate with 7500 real-time PCR System (Applied Biosystems). RNA relative expression was calculated as fold change using the comparative threshold cycle (*C*_T_) method (2^−ΔΔ*C*^_T_) with GAPDH or U6 serving as the internal control gene.

### Western blotting analysis

Cells were collected and lysed using RIPA protein extraction reagent (Beyotime, Beijing, China) supplemented with a protease inhibitor cocktail (Roche, Pleasanton, CA, U.S.A.) and phenylmethylsulfonyl fluoride (Roche). The concentrations of protein samples were detected using a BCA Protein assay kit (Beyotime). Equal amounts of protein extracts were separated by 10% SDS/PAGE gels electrophoresis, then transferred to PVDF membranes. The membranes were blocked for 1 h in Tris-buffered saline (TBS) containing 5% nonfat milk and incubated with primary antibodies at 4°C for 12 h. Antibodies against E-cadherin (#3195), N-cadherin (#5246), and Vimentin (#5741) were purchased from Cell Signaling Technology (Danvers, MA, U.S.A.). GAPDH signal was used as a control. Membranes were then incubated with goat anti-mouse or anti-rabbit secondary antibody (Beyotime, Shanghai, China) and visualized using enhanced chemiluminescence (ECL, Thermo Scientific, Shanghai, China).

### Wound-healing assay and cell transwell invasion assay

Cells were seeded on to six-well plates and cultured overnight. Wounds were created by scratching cell layer with a sterile 200-μl plastic pipette tips and washed with culture medium. Cells were further cultured with medium containing 1% FBS in 48 h, images were acquired at different time points by a microscope (Olympus, Tokyo, Japan) at 100× magnification. For the invasion assays, we used a 24-well transwell chamber (Corning: 8 μm) with the upper chamber coated with Matrigel (BD Bioscience). Cells (1 × 10^5^ cells in 100 μl serum-free medium) were seeded in the top chamber, 500 ml medium containing 10% FBS was placed into the lower chamber. After incubation for 24 h, cells on the upper membrane surface were wiped off using a cotton swab and the lower membrane surface was fixed with methanol, stained with 0.1% Crystal Violet, and counted in five random fields at 100× magnification.

### Luciferase reporter assays

The wild-type and mutant regions of *SPRY4-IT1* containing putative binding site with *miR-101-3p* were synthesized and constructed into the pGL3-control reporter vector (Promega, Madison, WI, U.S.A.). The introduction of mutations is listed in Figure 3A. The similar strategy was performed to assess the regulation relationship between ZEB1 and *miR-101-3p*. The wild-type fragment or three mutants of human *ZEB1* 3′UTR containing putative binding sites for miR-101-3p were synthesized. The introduction of mutations is listed in Figure 4A. HeLa and CaSki cells were harvested 24 h after transfection and the luciferase activity was detected by Dual Luciferase Reporter Assay Kit (Promega) according to the manufacturer’s instructions. Firefly, luciferase activities were normalized to *Renilla* luciferase activity. All experiments were performed in triplicate.

### Tumor xenografts experiments *in vivo*

All BALB/c nude mice (4 weeks old, female) were maintained under pathogen-free conditions and all procedures for the mouse experiments were approved by the Animal Care Committee of Shandong Provincial Hospital Affiliated to Shandong University. For the tumor xenografts experiments, stable HeLa and CaSki cells (5 × 10^6^, 100 μl) transfected with *SPRY4-IT1* shRNA or negative control were subcutaneously injected into mice (*n*=6 per group). Tumor growth was examined every 5 days after injection, and tumor volumes were calculated using the equation: length × width^2^ × 0.5. At 30 days post injection, mice were killed, and the tumors were excised, photographed and tissue sections were obtained for further study.

### Statistical analyses

All statistical analyses were performed with SPSS 20.0 (SPSS, Chicago, U.S.A.). Data were represented as mean ± standard deviation based on at least three repeats. Group difference was assessed using Student’s *t* test or one-way analysis of variance followed by post hoc Dunnett’s multiple comparisons test. *P*<0.05 was considered as statistically significant.

## Results

### Knockdown of SPRY4-IT1 inhibits cell proliferation, cell migration, cell invasion and EMT of CC *in vitro*

**Figure 1:**
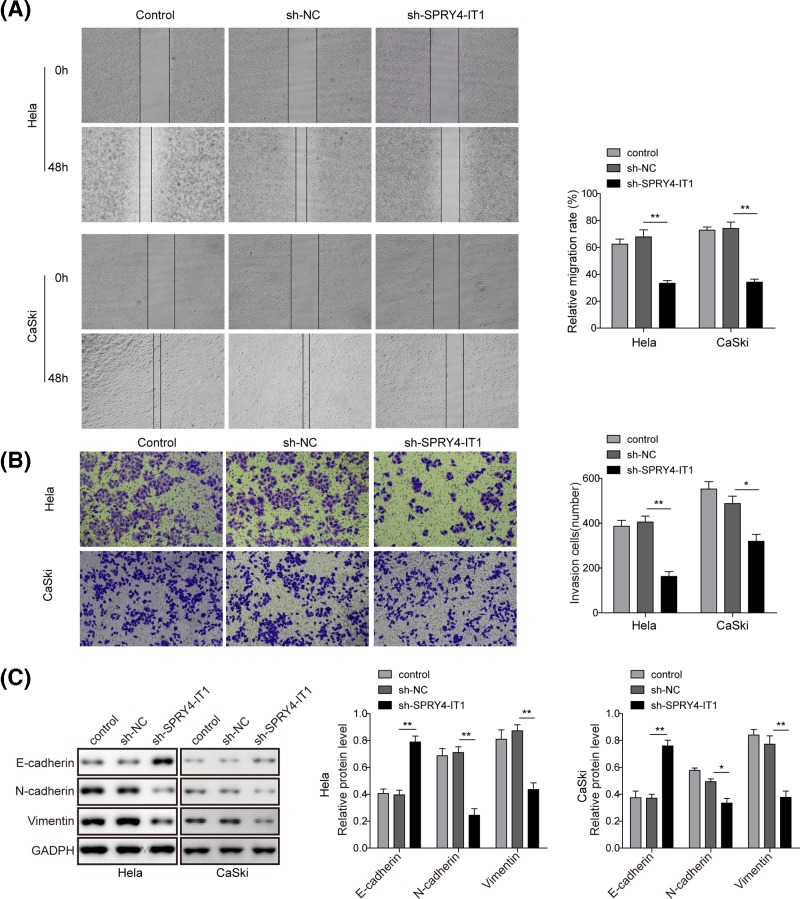
Knockdown of *SPRY4-IT1* inhibits cell proliferation, cell migration, cell invasion and EMT of CC *in vitro* (**A**) The migration potential of cells with *SPRY4-IT1* knockdown detected by wound healing assay. Comparison of cell motility of HeLa or CasKi cells transfected with sh-*SPRY4-IT1* or sh-NC was followed, starting at 0 h and continuing through to 48 h. Quantitation of wound healing was based on triplicate assays with error bars indicating the standard deviation (SD) of the mean. (**B**) The invasive potential of *SPRY4-IT1* knockdown in HeLa or CasKi cells detected by Transwell assay. Representative numbers of invading cells through the Matrigel were counted. (**C**) Expression of E-cadherin, N-cadherin and vimentin in HeLa or CasKi cells transfected with either sh-*SPRY4-IT1* or sh-NC analyzed by Western blot analysis. GADPH was used as a load control. Quantitative analysis of relative band intensity demonstrated the significant increase in E-cadherin and the decrease in N-Cadherin and vimentin protein levels upon *SPRY4-IT1* knockdown. Each bar represents triplicate analyses of mean ± SD, where the significant difference from the control group is represented by asterisk (^*^P<0.05, ^**^P<0.01).

The expression of *SPRY4-IT1* was significantly up-regulated in CC cell (*P*<0.05, Supplementary Figure S1A). Knockdown of *SPRY4-IT1* by shRNA inhibited the cell viability of HeLa and CaSki cells (*P*<0.05, Supplementary Figure S1B,C). Moreover, we further investigated the effect of knockdown of *SPRY4-IT1* on cell migration and invasion by an *in vitro* scratch assay and transwell assay, respectively. Knockdown of *SPRY4-IT1* indicated approximately 50% decrease in migration compared with sh-NC treatment in HeLa and CaSki cell lines (Figure 1A). The results of the invasion assay shows that knockdown of *SPRY4-IT1* significantly inhibits CC cell invasion in both cell lines (Figure 1B). Finally, we investigated the effects of *SPRY4-IT1* knockdown on the protein expression of E-cadherin, N-cadherin and vimentin (Figure 1C). Western blot analysis showed that knockdown of *SPRY4-IT1* up-regulated the expression level of the epithelial marker, E-cadherin, and down-regulated the mesenchymal maker, N-cadherin and vimentin in HeLa and CaSki cells. Taken together, the data suggest that down-regulation of *SPRY4-IT1* inhibits the migration and invasion of CC cells, which may be possibly via the EMT process.

### Knockdown of SPRY4-IT1 suppresses CC growth and metastasis *in vivo*

**Figure 2:**
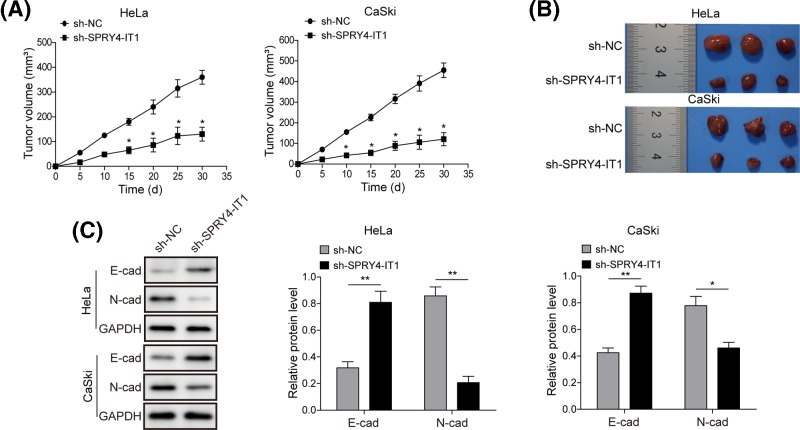
Knockdown of *SPRY4-IT1* suppresses CC growth and metastasis *in vivo* (**A**) Tumor volume curve of mouse upon sh-*SPRY4-IT1* or sh-NC treatment was analyzed. (**B**) Tumors collected from mice were exhibited. (**C**) Tumors developed from sh-*SPRY4-IT1* transfected cervical cell lines HeLa and CaSki showed higher E-cadherin protein levels and lower N-cadherin protein levels than tumors developed by negative control cells, determined by Western blot analysis. GADPH was used as a load control. Each bar represents triplicate analyses of mean ± SD, where the significant difference from the sh-NC is represented by ^*^P<0.05 and ^**^P<0.01.

To explore whether SPRY4-IT1 could affect tumorigenesis *in vivo*, HeLa and CaSki cells transfected with sh-SPRY4-IT1 or sh-NC were used in a nude mice xenograft model. Thirty days after injection, the tumors formed in sh-SPRY4-IT1 group were substantially smaller and lighter (*P*<0.05) than those in the sh-NC group (Figure 2A,B). Our qPCR analysis also confirmed the low expression of SPRY4-IT4 in tumor tissues of sh-SPRY4-IT4 group (*P*<0.05). Moreover, Western blot analysis showed that knockdown of *SPRY4-IT1* up-regulated E-cadherin expression and down-regulated N-cadherin expression in HeLa and CaSki cells, with the protein levels of E-cadherin was expressed at approximately two-fold higher while the level of N-cadherin was reduced by greater than 50% when compared with the sh-NC group (Figure 2C). Collectively, these data suggested that *SPRY4-IT1* contributes to CC metastasis *in vivo*, which may be partly via affecting EMT process.

### SPRY4-IT1 targets miR-101-3p to regulate the EMT process of CC

**Figure 3:**
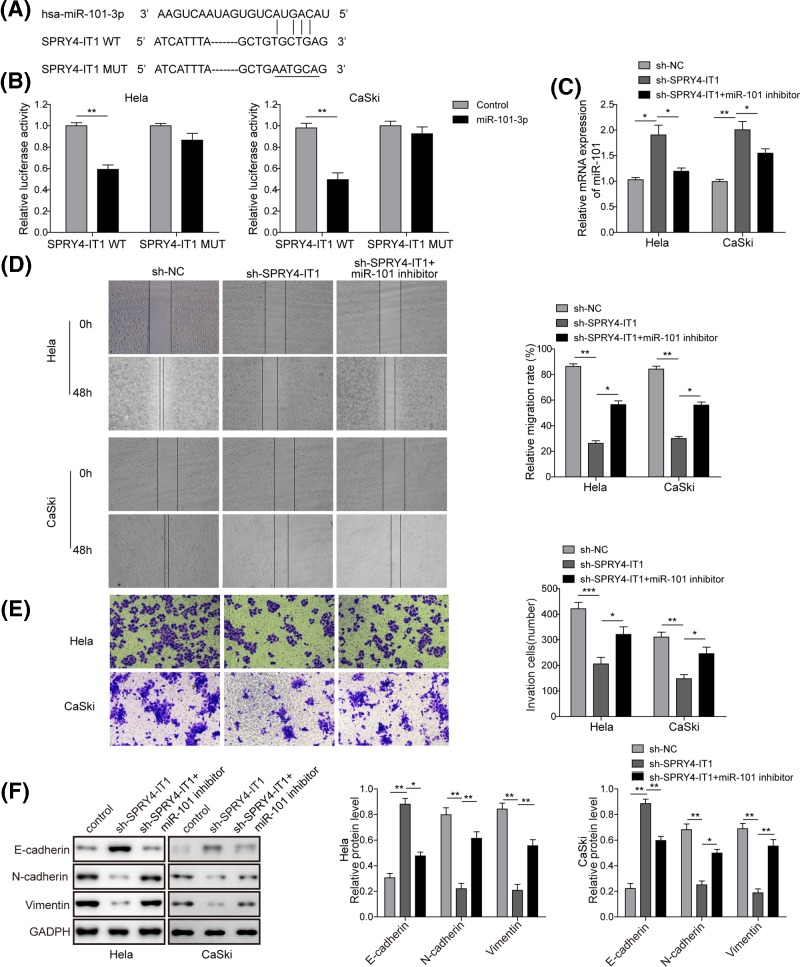
SPRY4-IT1 targets miR-101-3p to regulate the EMT process of CC (**A**) Sequence alignment of *miR-101-3p* with the putative binding sites within the wild-type or mutant regions of *SPRY4-IT1*. (**B**) The luciferase report assay demonstrated that overexpression of *miR-101-3p* reduced the intensity of fluorescence in HeLa and CaSki cells transfected with wild-type *SPRY4-IT1* vector, while it had on significant effect on mutant-type of *SPRY4-IT1*. (**C**) qPCR showed that knockdown of *miR-101-3p* with miR-101-3p inhibitor completely reversed the increased miR-101-3p mRNA expression induced by sh-*SPRY4-IT1* in HeLa and CaSki cells. (**D**) Wound healing assay showed that knockdown of *miR-101-3p* with *miR-101-3p* inhibitor partly reversed cell migration induced by sh-*SPRY4-IT1* in HeLa and CaSki cells. (**E**) Transwell assay showed that knockdown of *miR-101-3p* with *miR-101-3p* inhibitor partly reversed cell invasion induced by sh-*SPRY4-IT1* in HeLa and CaSki cells. (**F**) Western blotting analysis showed that knockdown of *miR-101-3p* with *miR-101-3p* inhibitor partly reversed the up-regulation of E-cadherin and down-regulation of N-cadherin and vimentin induced sh-*SPRY4-IT1* in HeLa and CaSki cells. ^*^P<0.05, ^**^P<0.01, ^***^P<0.001 compared with the sh-*SPRY4-IT1* group.

To identify the molecular mechanism by which *SPRY4-IT1* regulates the metastasis of CC, we first used a bioinformatics approach to predict the putative binding sites between *SPRY4-IT1* and *miR-101-3p* (Figure 3A). To identify the direct binding between *SPRY4-IT1* and *miR-101-3p*, the wild-type and mutant *SPRY4-IT1* fragment containing *miR-101-3p* binding site were synthesized and cloned into downstream of the luciferase reporter gene (pGL3-SPRY4-IT1-WT and pGL3-SPRY4-IT1-Mut) (Figure 3A). After co-trasfection of pGL3-SPRY4-IT1-WT or pGL3-SPRY4-IT1-Mut together with *miR-101-3p* mimics, luciferase activity was analyzed. The results revealed that the *miR-101-3p* significantly reduced the luciferase activities in pGL3-SPRY4-IT1-WT-treated HeLa and CaSki cells (40–50% reduction, *P*<0.01, Figure 3B), while the luciferase activities in pGL3-SPRY4-IT1-Mut-treated cells was not reduced by the expression of *miR-101-3p* (Figure 3B), suggesting that *SPRY4-IT1* is a direct target of *miR-101-3p*.

In support of these findings, we observed appproximately two-fold increase in the level of *miR-101-3p* mRNA in both HeLa and CaSki cells transfected with sh-*SPRY4-IT1*. Moreover, knockdown of *miR-101-3p* with *miR-101-3p* inhibitor reversed this increase induced by sh-*SPRY4-IT1* in CC cells (Figure 3C). Meanwhile, knockdown of *miR-101-3p* partly reversed cell migration and invasion ability inhibited by sh-*SPRY4-IT1* (Figure 3D,E). Additionally, we also found that *miR-101-3p* inhibitor efficiently reversed the up-regulation of E-cadherin protein level and the down-regulation of N-cadherin and vimentin protein levels induced by sh-*SPRY4-IT1* in HeLa and CaSki cells (Figure 3F). These data suggested that *SPRY4-IT1* modulated the EMT process by targets *miR-101-3p*.

### miR-101-3p targets ZEB1 to regulate the EMT process of CC

**Figure 4:**
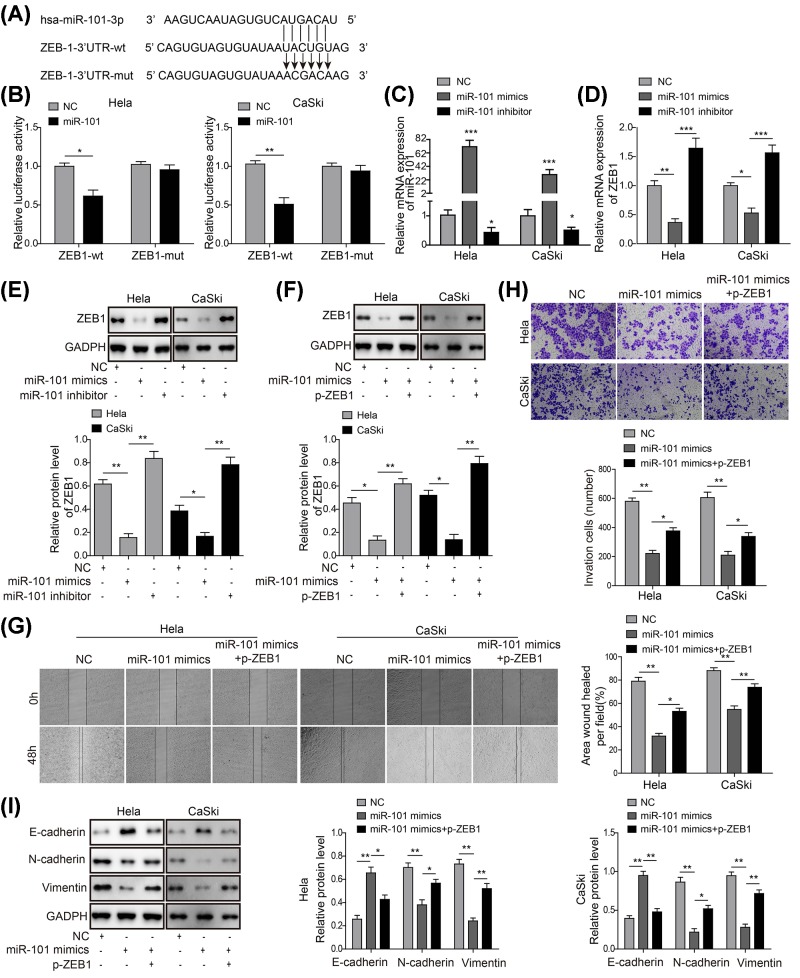
*miR-101-3p* targets ZEB1 to regulate the EMT process of CC (**A**) Sequence alignment of *miR-101-3p* with the putative binding sites within the wild-type or mutant regions of ZEB1. (**B**) The luciferase report assay demonstrated that overexpression of *miR-101-3p* reduced the intensity of fluorescence in HeLa and CaSki cells transfected with wild-type but not mutant-type ZEB1 vector. (**C**) qPCR showed that overexpression of *miR-101-3p* with *miR-101-3p* mimics up-regulated the levels of *miR-101-3p* mRNA, which was completely reversed by transfection of *miR-101-3p* inhibitor. (**D**) qPCR showed that overexpression of miR-101 with miR-101-3p mimics down-regulated the levels of ZEB1 mRNA, which was completely reversed by transfection of *miR-101-3p* inhibitor. (**E**) Western blotting analysis showed that *miR-101-3p* inhibitor completely reversed the suppression of ZEB1 induced by *miR-101-3p* minics in HeLa and CaSki cells. (**F**) Western blotting analysis showed that overexpression of ZEB1 by plasmid transfection (p-ZEB1) completely reversed the suppression of ZEB1 induced by *miR-101-3p* mimics in HeLa and CaSki cells. (**G**) Wound healing analysis showed that overexpression of ZEB1 by plasmid transfection (p-ZEB1) partly reversed cell migration induced by *miR-101-3p* mimics in HeLa and CaSki cells. (**H**) Transwell analysis showed that p-ZEB1 partly reversed cell invasion induced by *miR-101-3p* mimics in HeLa and CaSki cells. (**I**) Western blotting analysis showed that p-ZEB1 partly reversed the up-regulation of E-cadherin and down-regulation of N-cadherin and vimentin induced *miR-101-3p* mimics in HeLa and CaSki cells. ^*^P<0.05, ^**^P<0.01 and ^***^P<0.001 compared with the negative control (NC) group.

To find out if *ZEB1* was a direct target of *miR-101-3p*, 3′UTR fragment containing *miR-101-3p*-biding site (ZEB1 3′UTR-WT) and its mutant with substitution of six nucleotides (ZEB1 3′UTR-Mut) were synthesized and cloned downstream of the pGL3-control vector (Figure 4A). Dual luciferase reporter assay showed that *miR-101-3p* reduced the luciferase activities significantly in pGL3-ZEB1 3′UTR-WT-treated HeLa and CaSkis cells, while the luciferase activities in pGL3-ZEB1 3′UTR-Mut-treated cells was not reduced by the expression of *miR-101-3p* (Figure 4B).

To further verify the mechanism, we determined the levels of *miR-101-3p* and ZEB1 in HeLa and CaSki cells transfected with either *miR-101-3p* mimics or *miR-101-3p* inhibitor. We found that transfection of the *miR-101-3p* mimics in HeLa and CaSki cells caused an increase in the expression levels of *miR-101-3p*, while *miR-101-3p* inhibitor dramatically reduced the *miR-101-3p* expression (Figure 4C). Importantly, a greater than 50% decrease in the mRNA and protein levels of ZEB1 were observed in cells transfected with the *miR-101-3p* mimics and *miR-101-3p* inhibitor led to an opposite effect (Figure 4D,E). Moreover, we found overexpression ZEB1 by plasmid transfection (p-ZEB1) also reversed the suppression of ZEB1 induced by transfection of *miR-101-3p* mimics (Figure 4F). Overexpression of ZEB1 also partly reversed cell migration and invasion ability induced by *miR-101-3p* mimics (Figure 4G,H). Additionally, we also found that overexpreesion of ZEB1 efficiently reversed the up-regulation of E-cadherin protein level and the down-regulation of N-cadherin and vimentin protein levels induced by *miR-101-3p* mimics in HeLa and CaSki cells (Figure 4I). These data suggested that *miR-101-3p* modulated the EMT process partly through the regulation of ZEB1.

### SPRY4-IT1 targets miR-101-3p to regulate ZEB1 expression in the EMT process of CC

**Figure 5:**
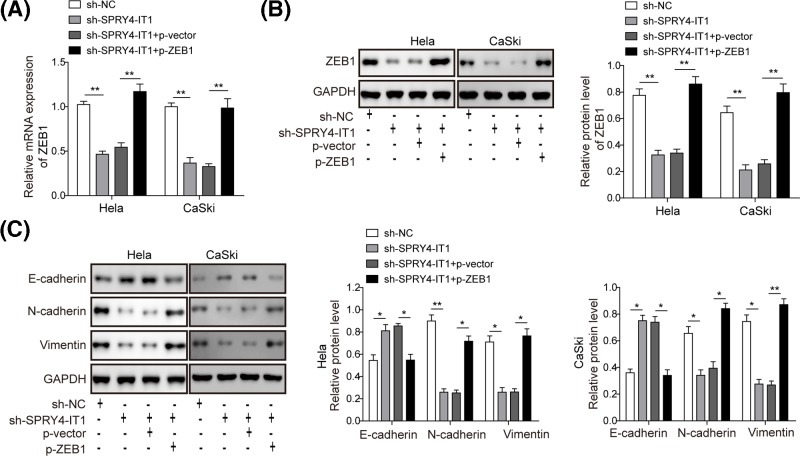
*SPRY4-IT1* targets *miR-101-3P* to regulate ZEB1 expression in the EMT process of CC (**A**) qPCR showed that overexpression of ZEB1 by plasmid transfection effectively reversed the reduction in *SPRY4-IT1* expression levels induced by sh-*SPRY4-IT1*. (**B**) Western blotting analysis showed that overexpression of ZEB1 effectively reversed the reduction in ZEB1 protein levels induced by sh-*SPRY4-IT1*. (**C**) Western blotting analysis showed that overexpression of ZEB1 efficiently reversed the up-regulation of E-cadherin protein level and the down-regulation of N-cadherin and vimentin protein levels induced by sh-*SPYR4-IT1* in HeLa and CaSki cells. ^*^P<0.05, ^**^P<0.01 compared with the sh-NC group or sh-*SPRY4-IT1*+p-vector group.

Considering the regulatory relationship between *SPRY4-IT1/miR-101-3p*, and *ZEB1/miR-101-3p*, we hypothesized that *SPRY4-IT1* might regulate the expression levels and functions of ZEB1. To validate this hypothesis, we treated HeLa and CaSki cells with sh-*SPRY4-IT1* with or without ZEB1 overexpression vector and determined the expression levels of *SPYR4-IT1* and ZEB1. We found that the *ZEB1* expression levels were significantly reduced in HeLa and CaSki cells transfected with sh-*SPRY4-IT1* compared with sh-NC treatment. But overexpression of ZEB1 by plasmid transfection effectively restored the decreased *ZEB1* expression induced by sh-*SPRY4-IT1* (Figure 5A). Similarly, the ZEB1 protein levels were significantly reduced in HeLa and CaSki cells transfected with sh-*SPRY4-IT1* and overexpression of ZEB1 effectively reversed this decrease, as determined by Western blotting analysis (Figure 5B). Moreover, we found that overexpression of ZEB1 efficiently reversed the up-regulation of E-cadherin protein level and the down-regulation of N-cadherin and vimentin protein levels induced by sh-*SPYR4-IT1* in HeLa and CaSki cells (Figure 5C). Taken together, our data suggested that *SPRY4-IT1* regulate ZEB1 expression in the EMT process of CC.

## Discussion

Emerging evidence has revealed a key regulatory role of lncRNAs in tumorigenesis, although only a few functional lncRNAs have been identified in CC [4]. Identification of dysregulated lncRNAs will enhance our knowledge of lncRNAs function in the progression and metastasis of CC and could be used as new diagnostic or therapeutic targets. In the present study, we confirmed that shRNA-mediated *SPRY4-IT1* knockdown induced significant inhibition of cell migration, and invasion ability in CC both *in vitro* and *in vivo* via regulating the EMT process. Mechanistically, we showed that *SPRY4-IT1* directly interacted with *miR-101-3p* at recognized sites. *SPYR4-IT1* exerted its oncogenic function on CC in large part due to its role to function as an *miR-101-3p* sponge, and subsequently initiate ZEB1 signaling pathway. These findings indicate that *SPRY4-IT1* could function as an oncogenic regulatory hub and may be useful as a novel prognostic or progression marker for CC.

Although *SPRY4-IT1* can promote migration and invasive phenotype of CC cells, the underlying mechanism is still elusive. Previous study showed that *SPRY4-IT1* is decreased in gastric cancer, and the inhibitory effects of *SPRY4-IT1* on cell migration and invasion were partly associated with EMT progression [20]. Our data show that knockdown of *SPRY4-IT1* indeed reduced the cell motility and invasion *in vitro*. And in our tumor xenograft models, *SPRY4-IT1* knockdown significantly reduced the tumorigenesis ability of HeLa and CaSki cells *in vivo*. EMT is a key step toward cancer metastasis, a biological process where epithelial cells lose their polarity and undergo transition into a mesenchymal phenotype. Loss of E-cadherin expression is a hallmark of EMT process in epithelial cancer including CC, which has been found to increase cancer cell invasion and metastasis [6]. In our research, we further demonstrated that *SPRY4-IT1* promoted CC cell migration and invasion partially via regulating EMT progression, as inhibition of *SPRY4-IT1* induced differential enforcement of E-cadherin and reduction in N-cadherin and vimentin both in CC cells and in mice tumor tissues with *SPRY4-IT1* knockdown.

One of the mechanisms that lncRNAs function through is to play regulatory roles by acting as target genes for specific miRNAs, then targeting other terminal mRNAs [18]. In bladder cancer, *SPRY4-IT1* mediates EZH2 expression by acting as an miR-101b sponge [22]. Similarly, our results confirmed that *SPRY4-IT1* could act as an upstream regulator for miR-101-3p and promoted migration and invasion in CC cells. To clarify whether *SPRY4-IT1* functions as a sponge for *miR-101-3p* in CC, bioinformatics was used to analyze the potential miRNA binding sites contained in *SPYR4-IT1*. Luciferase reporter assay and qPCR showed that *SPRY4-IT1* could positively regulate the EMT progression at post-transcriptional level through targeting *miR-101-3p* in CC.

Emerging evidence has demonstrated that several miRNAs could target the 3ʹ-UTR of the ZEB1, a key transcription factor of EMT, and post-transcriptionally regulate its expression in cancers [5]. Previous study has revealed that *miR-101* could directly target ZEB1 to repress ZEB1 expression at post-transcriptional level in breast cancer [16]. While opposite effects of ZEB1 and *miR-101-3p* in CC whereby ZEB1 functions as an oncogene [12], and *miR-101-3p* as a tumor suppressor [15] have also been demonstrated, their direct interaction in CC has not yet studied. Our results also demonstrate that *miR-101-3p* acts as a tumor suppressor in CC. Overexpression of *miR-101-3p* inhibited cell proliferation and invasion of CC cells *in vitro*. This may be partially through the regulation of EMT, as overexpression of *miR-101-3p* also increased the levels of E-cadherin and decreased the levels of N-cadherin and vimentin CC cells. Moreover, the enforced expression of *miR-101-3p* reduced the ZEB1 expression and *miR-101-3p* inhibitor, as expected, promoted ZEB1 expression at protein level. Furthermore, ZEB1 activation was responsible for EMT triggered by overexpression of *miR-101-3p*, as overexpression of ZEB1 restored epithelial phenotype and migration and invasion in CC cells induced by enhanced *miR-101-3p* expression. Hence, our work verified that ZEB1 is one of the target genes of *miR-101-3p*, which is involved in cell migration and invasion in CC by regulating EMT. Further exploration showed that *SPRY4-IT1* and ZEB1 have shown a positive correlation in CC cells, and knockdown of *SPRY4-IT1* decreased ZEB1 at protein levels *in vitro*. In addition, overexpression of ZEB1 could partially rescue the decreased migration and invasion by *SPRY-IT1* knockdown. These results indicate that *SPRY4-IT1* could regulate ZEB1 via *miR-101-3p*.

In summary, we identify that *SPRY4-IT1* acts as an oncogene in CC, competitively binds to *miR-101-3p* and subsequently up-regulates the expression of its target gene ZEB1 to promote the migration and invasion of CC cells. This SPYR4-IT1/miR-101-3p/ZEB1 regulatory network may shed light on tumorigenesis in CC and may be valuable for the development of novel diagnostic and treatment approaches for CC.

## Supporting information

**Supplementary Figure:** 

## Abbreviations

CC: cervical cancer
EMT: epithelial–mesenchymal transition
FBS: fetal bovine serum
GAPDH: glyceraldehyde-3-phosphate dehydrogenase
LncRNA: long non-coding RNA
MET: mesenchymal-epithelial transition
miRNA: microRNA
qPCR: quantitative real-time PCR
shRNA: short hairpin RNA
SPRY4: Sprouty RTK Signaling Antagonist 4
SPRY4-IT1: SPRY4 intronic transcript 1
UTR: untranslated region
ZEB1: Zinc finger E-box-binding homeobox 1
