# Correction: Long non-coding RNA SPRY4-IT1 promotes epithelial-mesenchymal transition of cervical cancer by regulating the miR-101-3p/ZEB1 axis

**DOI:** 10.1042/BSR-2018-1339_COR

**Published:** 2024-07-31

**Keywords:** cervical Cancer, EMT, Long non-coding RNA, metastasis, SPRY4-IT1

The authors of the original article “Long non-coding RNA SPRY4-IT1 promotes epithelial–mesenchymal transition of cervical cancer by regulating the miR-101-3p/ZEB1 axis” (10.1042/BSR20181339) would like to correct Figure 4 in their paper. The authors state that due to incorrect image exporting and selection during figure preparation, the Figure 4H CaSki miR-101 mimics+p-ZEB1 image was accidentally used that originated from the CaSki miR-101 mimics group.

**Figure 4:**
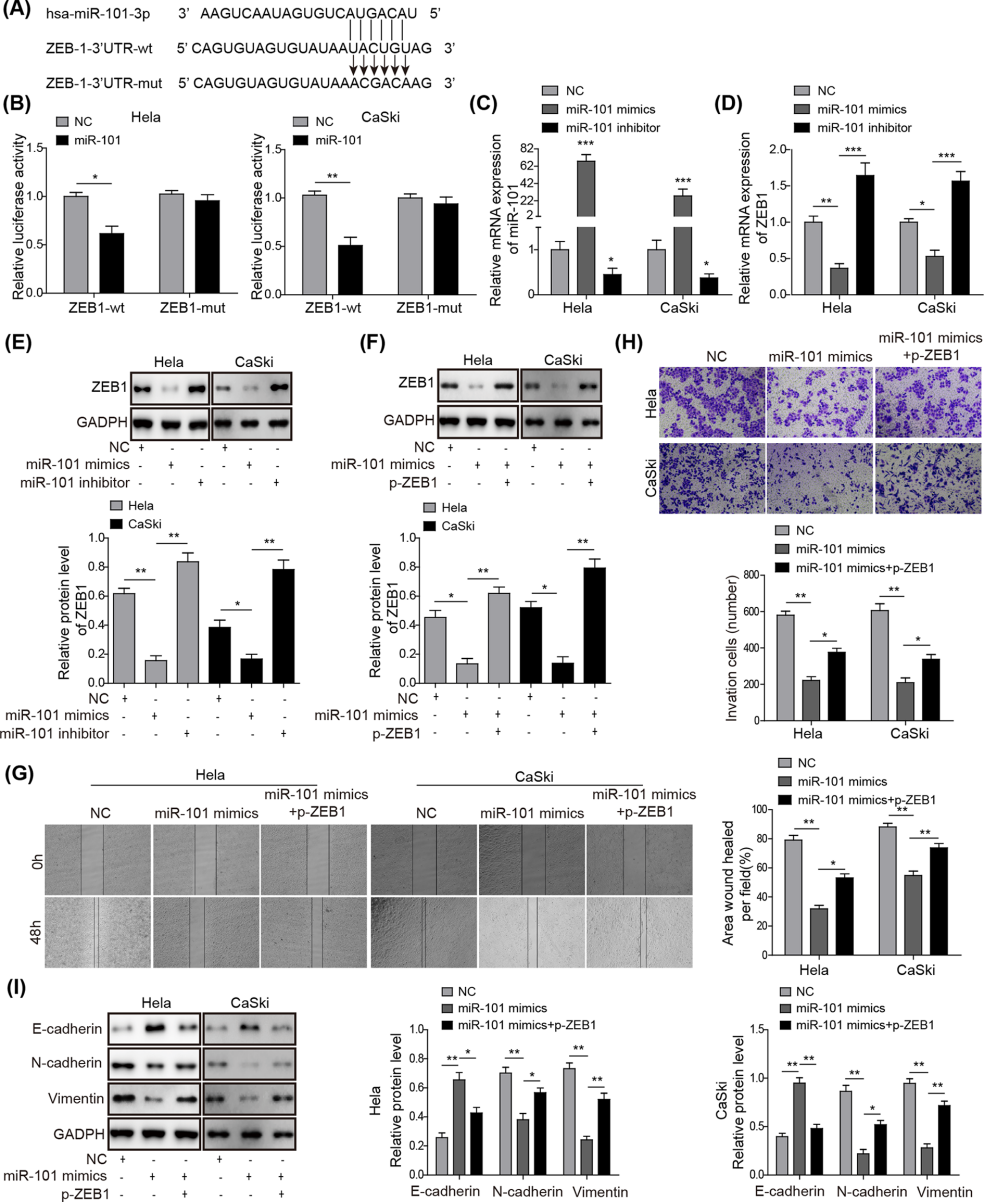
miR-101-3p targets ZEB1 to regulate the EMT process of CC

The requested correction has been assessed and agreed by the Editorial Board. The authors declare that the correction does not change the results or conclusions of their paper. The corrected version of Figure 4 is presented here.

