# Supplementary figures and images for: Long non-coding RNA SPRY4-IT1 promotes epithelial–mesenchymal transition of cervical cancer by regulating the miR-101-3p/ZEB1 axis
Source: Biosci Rep. 2019 Jun 4;39(6):BSR20181339. doi: 10.1042/BSR20181339 (PMC6549091; doi:10.1042/BSR20181339)

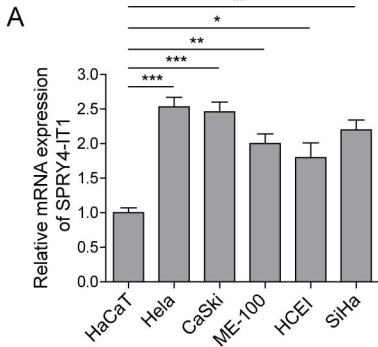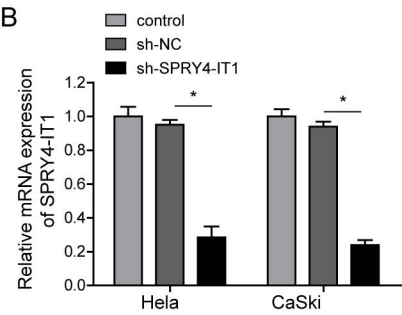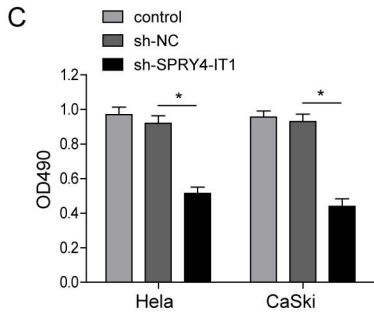

Supplement: Supplementary file 1 [file bsr20181339_Supp1.pdf]
